# A Case of Paradoxical Embolism Causing Anterior Spinal Cord Syndrome and Acute Myocardial Infarction Following the Intradiscal Oxygen-Ozone Therapy

**DOI:** 10.3389/fneur.2019.00137

**Published:** 2019-02-22

**Authors:** Runcheng He, Qing Huang, Xinxiang Yan, Yunhai Liu, Jie Yang, Xiaobin Chen

**Affiliations:** ^1^Department of Neurology, Xiangya Hospital, Central South University, Changsha, China; ^2^National Clinical Research Center for Geriatric Disorders, Changsha, China; ^3^Hunan Clinical Research Center of Cerebrovascular Disease, Changsha, China; ^4^Key Laboratory of Hunan Province in Neurodegenerative Disorders, Central South University, Changsha, China; ^5^Department of Cardiology, Xiangya Hospital, Central South University, Changsha, China

**Keywords:** intradiscal oxygen–ozone therapy, anterior spinal cord syndrome, myocardial infarction, patent foramen ovale, air embolism

## Abstract

We report a case of a 66-year-old female who burst into flaccid paralysis of the lower extremities, accompanied by loss of pain and temperature sensation below T4 level, during an oxygen–ozone injection for disc herniation. Half an hour later, she suffered from chest pain. Magnetic resonance imaging (MRI) showed long segment hyperintensity in the thoracic spinal cord from T2 to 10 level on sagittal T2-weighted images (T2WI). The electrocardiogram (ECG) showed ST-segment elevation in V1–V6 leads. She was diagnosed with spinal cord infarction and ST-elevation myocardial infarction (STEMI). Transthoracic echocardiography with saline contrast showed existence of a large patent foramen ovale (PFO) correlating with the detection of massive microbubbles in the left atrium. We discuss the potential role of paradoxical embolism in spinal cord infarction and myocardial infarction.

## Introduction

**Figure 1:**
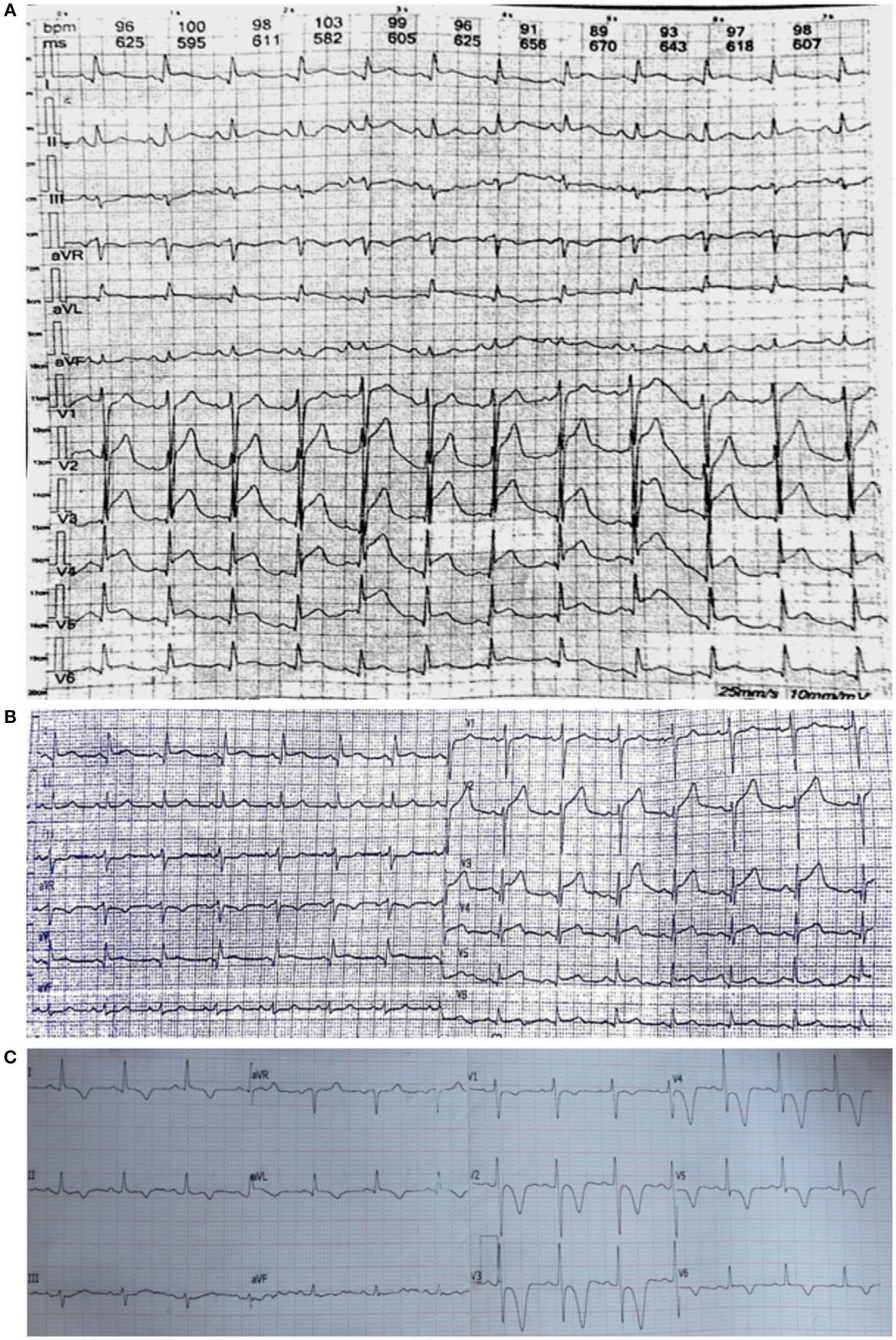
**(A)** Post-procedure ECG shows significant ST elevations in leads V1–V6. **(B)** After 2 h of treatment, the ST segments have reduced. **(C)** One month later, ECG shows T wave inversion with ST depression in leads V1–V6.

A 66-year-old female accountant was admitted to spine surgery department because of recurrent low back pain. She was diagnosed with lumbar disc herniation at L4/L5 and L5/S1 levels by Magnetic resonance imaging (MRI). Before procedure, she could perform daily activities independently without hypertension and coronary heart disease. In March 2018, she underwent a percutaneous intradiscal injection of oxygen–ozone at a local hospital. Under the CT scan guidance, the puncture needle was placed in the L4–L5 disc of the patient lying in prone position. After the injection of 8 ml of oxygen-ozone mixture, she complained of weakness and loss of sensation in the bilateral lower limbs. At this moment, intradiscal injection of gas was stopped immediately. A comprehensive neurological exam demonstrated complete flaccid paralysis of both lower extremities with motor power of 0/5 on the Medical Research Council (MRC) scale and absence of deep tendon reflexes. Sensation to pinprick and temperature were impaired below T4 level, but light touch and proprioception in the lower extremities were intact. The examinations of cranial nerves and upper extremities were normal. Five minutes later, she complained of acute chest pain. The electrocardiogram (ECG) showed ST-segment elevation in leads V1–V6 (Figure 1A), suggesting an acute large anterior wall ST-segment elevation myocardial infarction. Immediately, she was treated with intravenous heparin 5,000 U, oral aspirin 300 mg and clopidogrel 300 mg. Laboratory workup revealed creatine kinase MB (CKMB) 69 U/L, troponin I 3.049 ng/mL. D-dimmer tests were within normal. However, subsequent coronary digital subtraction angiogram (DSA) did not detect vascular abnormality. One hour later, the patient showed clinical relief of chest discomfort with progressive normalization of ECG (Figure 1B).

**Figure 2:**
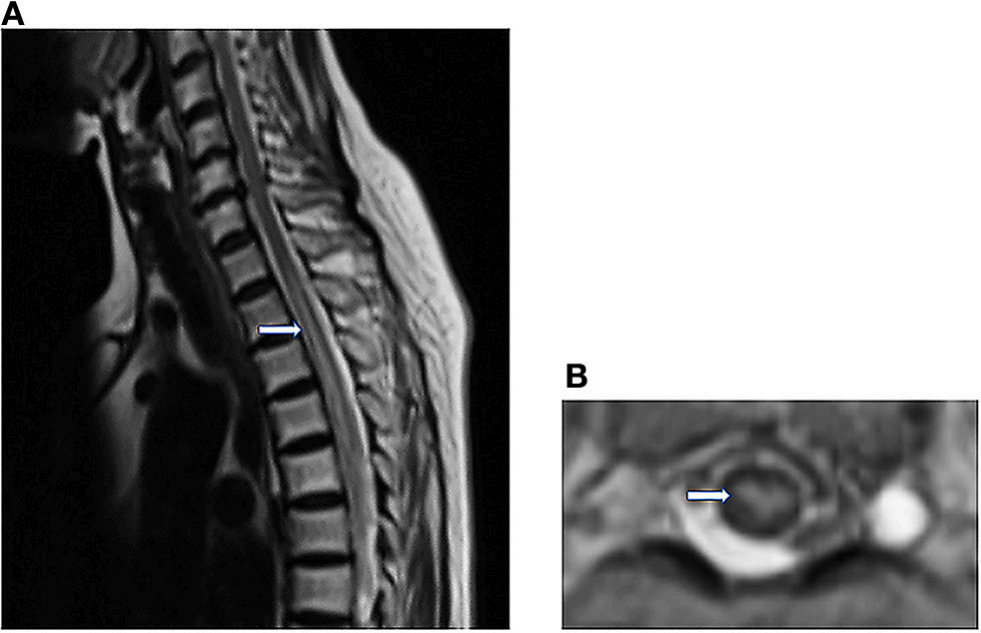
**(A)** Sagittal T2WI reveals abnormally Linear hyperintensity in the thoracic cord, extending from T2 to 10 level. **(B)** Axial T2WI shows the high signal intensity in the anterior horn of the gray matter (owl's eye appearance) at the T8 level.

**Figure 3:**
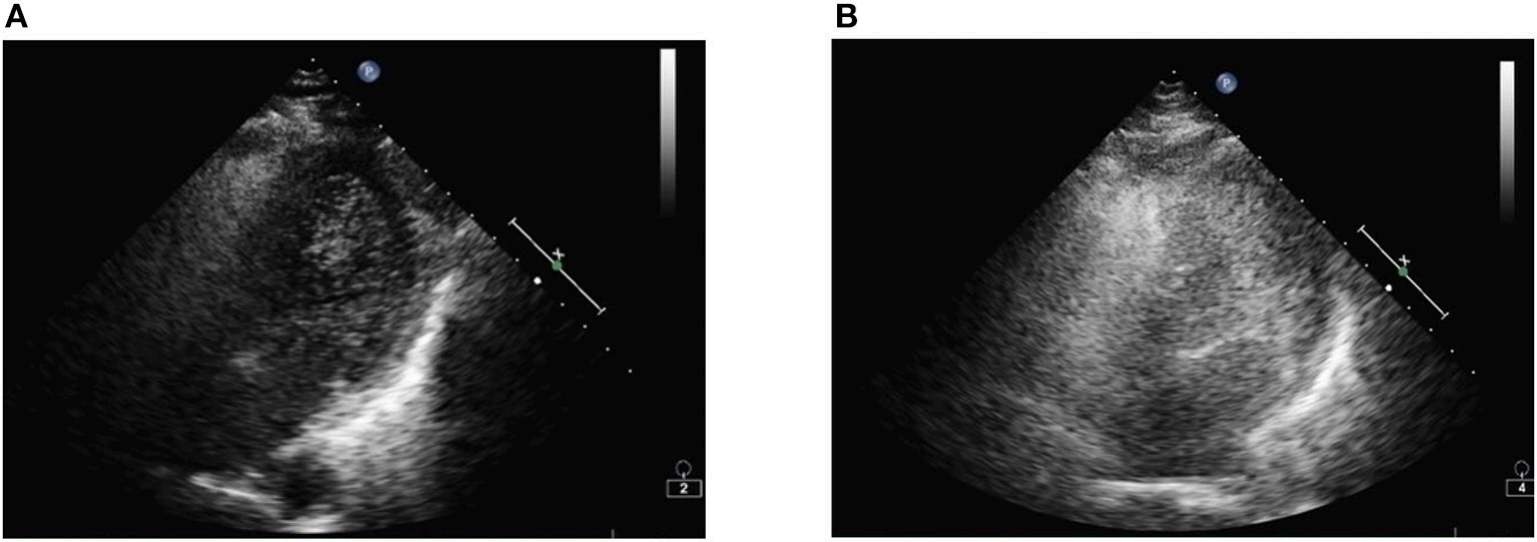
**(A)** After injection of agitated saline, microbubbles are visible in the left ventricle by transthoracic echocardiography. **(B)** After the Valsalva maneuver, a large volume of microbubbles is filling the left ventricular and subsequently the left ventricular.

Three days after the onset of paraplegia, she was admitted to our hospital. The MRI revealed a hyperintensity in the thoracic cord from T2 to 10 level on T2-weighted images (T2WI) (Figures 2A,B). Computed tomography angiography (CTA) disclosed no aortic disease or vertebral stenosis. Transthoracic echocardiography with saline contrast showed a right-to-left shunt with massive microembolic signals in the left atrium during the resting state (Figure 3A) and Valsalva maneuver (Figure 3B), indicating the presence of a large patent foramen ovale (PFO). Vascular ultrasound didn't detect venous thrombosis in the four extremities. Therefore, she was diagnosed with anterior spinal cord syndrome and ST-elevation myocardial infarction (STEMI), which were most likely caused by air embolism. She received conservative treatment, including hyperbaric oxygen, oral aspirin 100 mg, injection of alprostadil and mouse nerve growth factor, and rehabilitation training. At the same time, she also received bladder catheterization.

**Figure 4:**
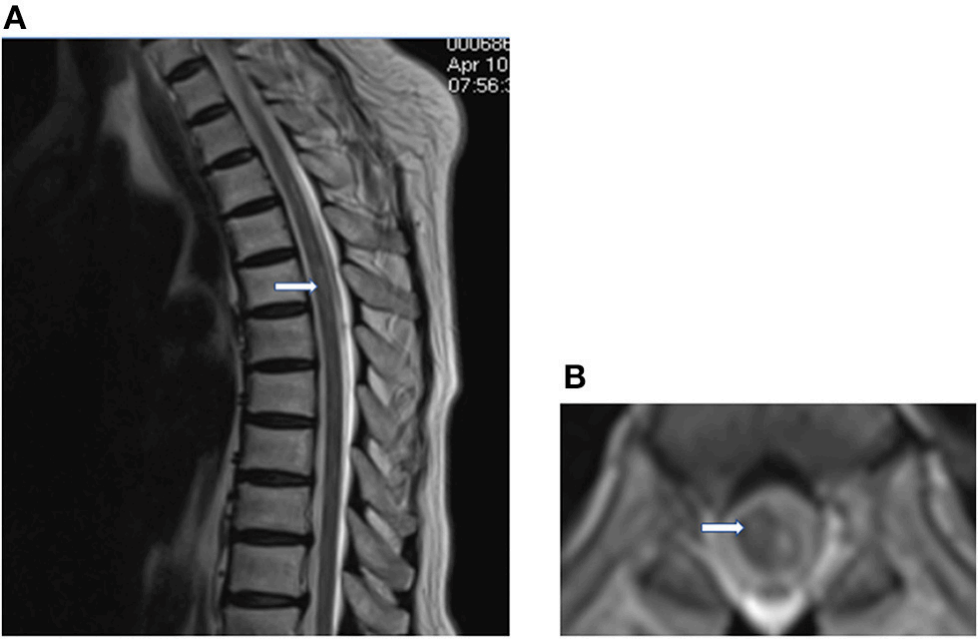
One month later, sagittal **(A)** and axial T2WI **(B)** shows a decrease in the hyperintense region.

**Figure 5:**
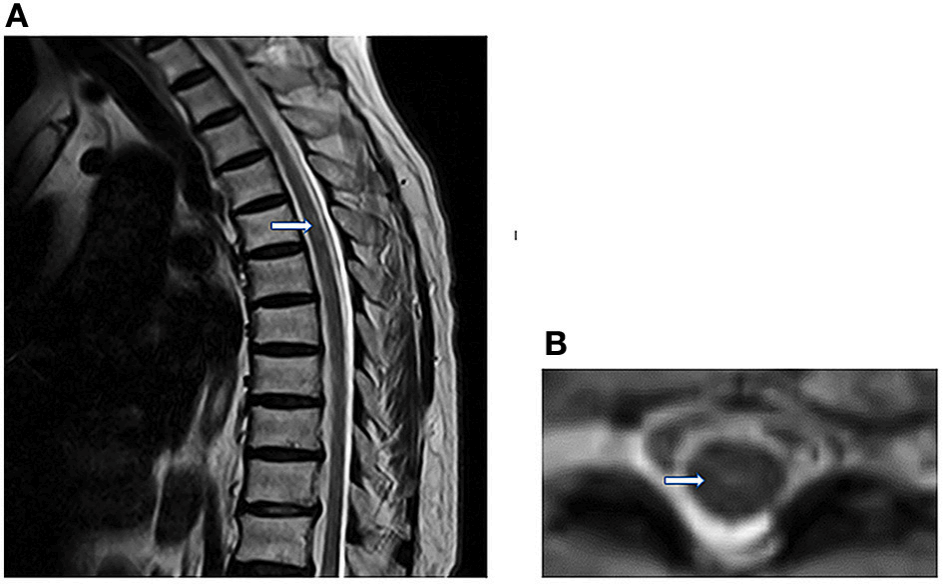
Nine months later, sagittal **(A)** and axial T2WI **(B)** showed that the lesion volume has significantly reduced.

During the follow-up, 1 month later, we observed her clinical improvement with motor power of 2/5 in right lower limb and 1/5 in left lower limb, along with hyperreflexia and abnormal reflexes in the bilateral lower limbs. She had no recurrence of chest pain with normal cardiac enzymes and troponin I. The ECG showed T inversion in V1–V6 (Figure 1C). MRI showed a decrease in the size of spinal cord lesion (Figure 4). Nine months later, she reported that she was recovering from paraplegia and was able to stand with a cane. Motor power returned to 4/5 in right lower extremity and 3/5 in left. Abnormal reflexes were unchanged. Follow-up MRI showed the lesion volume on T2WI has significantly reduced (Figure 5).

## Background

Intradiscal oxygen–ozone therapy is a widely used minimally invasive approach for the treatment of disc herniation with the best cost/benefit ratio (1, 2). Oxygen–ozone therapy was first advocated by Italian doctor Verga to treat lumbar and leg pain in 1988. Because around 55% of the population in western countries have suffered from low back pain associated with radicular syndromes (3). The main mechanisms of ozone therapy include two aspects. On one hand, ozone can inhibit the synthesis and release of inflammatory mediators, thereby reducing chemical radiculitis (4). On the other hand, it can react with the mucopolysaccharides of nucleus pulposus in the intervertebral disc, leading to their rupture and shrinkage of the herniated material. As a result, this treatment can reduce nerve root compression due to herniated disc material and relieve herniated disc-related pain (5, 6). A large number of studies have demonstrated that the oxygen–ozone therapy had achieved satisfactory results (7). Nowadays, the low costs of this therapy and the 75–80% success rate has made it a popular method for percutaneous treatment of disc herniation in several countries, including China and European countries (8, 9).

Anterior spinal cord syndrome is a relatively rare nervous system disease, accounting for only 5–8% of myelopathies (10). This syndrome is characterized by paralysis of the extremities, dissociated sensory loss and bowel and bladder dysfunction (11). The reason for this is the damage or obstruction of the anterior spinal artery, which originates from the vertebral artery and provides the major blood supply to the central and peripheral parts of the anterior two-thirds of the spinal cord. The most common location is the thoracic segment. Because anterior spinal artery has the narrowest diameter in this region (12). Prior to the present report, there have been few previous reports on cases of the anterior spinal cord syndrome associated with oxygen–ozone therapy. We herein report a case of spinal cord infarction and STEMI after intradiscal oxygen–ozone therapy.

## Discussion

Spinal cord infarction accounts for only 0.3–1% of stroke cases (13). This patient was diagnosed with spinal cord infarction due to the acute course, typical clinical manifestations, and imaging evidences of anterior spinal cord syndrome. Several researches have reported that hyperlipidemia, diabetes, hypertension, atherosclerotic lesions, and a history of cerebral infarction are high risk factors for spinal cord infarction (13–16). However, there were no common risk factors for spinal cord infarction. Furthermore, aortic and vertebral dissections or stenosis were ruled out by CTA, and vasculitis was negative for autoantibodies. The only risk factor we found is PFO, which is associated with embolism. As a result, the accident is most probably caused by air embolus produced by oxygen–ozone injection and subsequently entering arterial circulation. It has been demonstrated that neurologic deficits and cardiac arrest are common clinical symptoms of arterial air embolism (17–19). The initial clinical manifestation of this patient was the relatively rare anterior spinal cord syndrome caused by paradoxical embolism of anterior spinal artery (20).

Acute chest pain is a common presenting symptom of acute coronary syndrome (ACS), including ST elevation myocardial infarction (STEMI) and non-ST elevation ACS (21). The essential risk factors for ACS are hypertension, diabetes, dyslipidemia, obesity, smoking, and positive family history (22–24), however, these risk factors were not present in this patient. The coronary DSA showed no significant stenosis or occlusion in the coronary artery. However, it has been reported that paradoxical embolism can result in ACS as well (25). Air embolism is a rare cause of ACS, but even a miniscule amount of air embolus can also disrupt coronary blood flow leading to devastating consequences (26). Air embolism generally occurs in the right coronary artery, which applies for patient in the supine position. In this case, air embolism entered the left coronary artery because the patient underwent oxygen–ozone injection in the prone position.

Although intradiscal oxygen–ozone therapy is a low-risk procedure. Air embolism, as a rare complication, has been a matter of serious concern. As we all know, a portion of oxygen-ozone gas can enter the spinal canal from the chasm of intervertebral disc when it is injected into the disc. With the gradual increase of the leaking gas, a certain pressure gradient will drive gas into the venous circulation through the venous plexus, which are very abundant in the spinal canal. Sometimes a patient's intrathoracic pressure may increase dramatically due to pain during injection, facilitating a transient rise in atrial pressure and leading to a right-to-left shunt. Then gas embolus can reach the anterior spinal artery and coronary artery through the PFO (27). Assuming there is no right-to-left shunt, the venous gas embolus will lodge in the pulmonary vasculature and cause pulmonary artery hypertension. In addition, if an anatomic cardiac defect is present, venous gas embolus can also enter to arterial circulation through right-to-left shunt, such as PFO and fistulous tract. Eventually, gas embolus will affect systemic arterial blood flow and cause end-organ ischemia or infarction.

Air embolism is a potential complication of intradiscal oxygen–ozone therapy. In previous studies, some researches indicated that hyperbaric oxygen therapy (HBOT) will have an essential role in the treatment of air embolism by reducing bubble volume and improving tissue oxygenation (18, 28, 29). Subsequently, gas embolus can be dissolved in blood because of the effect of surface tension. In addition, improving microcirculation may also have a beneficial effect on air embolism.

## Concluding Remarks

In recent years, intradiscal oxygen–ozone therapy has been playing an important role in treating disc herniation. But it can also lead to some complications, such as paradoxical arterial embolism, in patients with right-to-left shunt. To avoid this complication, patients should be screened for right-to-left shunt prior to intradiscal oxygen–ozone therapy. If right-to-left shunt is detected, it is best not to undergo intradiscal oxygen–ozone therapy for prevention of air embolism.

## Ethics Statement

This study was carried out in accordance with the recommendations of Ethics Committee of Xiangya Hospital of Central South University. The protocol was approved by the Ethics Committee of Xiangya Hospital of Central South University. The subject gave written informed consent in accordance with the Declaration of Helsinki. And the she gave written informed consent for publication of this report as well.

## Author Contributions

RH drafting the manuscript. QH, XY, and XC analysis and interpretation of data. YL revising the manuscript. JY study concept and design.

### Conflict of Interest Statement

The authors declare that the research was conducted in the absence of any commercial or financial relationships that could be construed as a potential conflict of interest.
